# Global Abortion Policies Database: a new approach to strengthening knowledge on laws, policies, and human rights standards

**DOI:** 10.1186/s12914-018-0174-2

**Published:** 2018-09-12

**Authors:** Brooke Ronald Johnson, Antonella Francheska Lavelanet, Stephanie Schlitt

**Affiliations:** 1 Department of Reproductive Health and Research and UNDP-UNFPA-UNICEF-WHO-World Bank Special Programme of Research, Development and Research Training in Human Reproduction (HRP), World Health Organization, 20 Avenue Appia, CH-1211 Geneva 27, Switzerland; 2 UNDP-UNFPA-UNICEF-WHO-World Bank Special Programme of Research, Development and Research Training in Human Reproduction (HRP), World Health Organization, 20 Avenue Appia, CH-1211 Geneva 27, Switzerland

**Keywords:** Abortion laws, Abortion policies, Abortion guidelines, Abortion human rights standards

## Abstract

**Background:**

The Global Abortion Policies Database (GAPD), launched in June 2017, provides a verifiable, comprehensive, nuanced approach to information and data sources on abortion law and policy. Abortion laws, policies, and guidelines from United Nations (UN) and World Health Organization (WHO) Member States are juxtaposed to information and recommendations from WHO safe abortion guidance, national sexual and reproductive health indicators, and relevant UN human rights bodies’ concluding observations to countries.

**Main body:**

The Global Abortion Policies Database aims to increase transparency of information and accountability of states for the protection of individuals’ health and human rights. The database presents current information on abortion laws and policies that goes beyond categories of lawful abortion to include information on additional access requirements, service provision, conscientious objection, and penalties. Wide-ranging variations among countries’ legal requirements and criminal penalties raise questions about the evidentiary and human rights basis for abortion laws and policies. Source documents found in the database highlight that in many jurisdictions legal and policy guidance is either non-existent, not clear, or conflicting. By juxtaposing a jurisdiction’s abortion laws and policies to relevant WHO guidance and by facilitating comparisons of countries’ sexual and reproductive health indicators, the database can enable deep policy analysis of states’ obligations to meet the health needs and human rights of individuals in the context of abortion. Policy analysis in the context of authoritative guidance on human rights standards can enable health and rights advocates to hold governments accountable for respecting, protecting, and fulfilling individuals’ human rights.

**Conclusion:**

The GAPD is a comprehensive tool that can be used to strengthen knowledge, inform law and policy research to generate evidence on the impact of laws and policies in practice, and facilitate greater awareness of the many challenges to creating enabling policy environments for safe abortion.

## Background

The Global Abortion Policies Database (GAPD), launched in June 2017, is a policy tool designed to increase the transparency of global abortion laws and policies and state accountability for the protection of women’s and girls’ health and human rights [1, 2]. The GAPD contains abortion laws, policies, standards and guidelines for United Nations (UN) and World Health Organization (WHO) Member States^1^ including selected subnational and dependency jurisdictions.

The GAPD presents information on abortion laws and policies that goes beyond categories of lawful abortion to include information on additional legal requirements such as third-party authorisation and mandatory waiting periods, clinical and service-delivery aspects of abortion care, conscientious objection, and penalties. The GAPD only includes data that can be linked to a downloadable source document^2^. National sexual and reproductive health indicators and UN Treaty Monitoring Body concluding observations and Special Procedures reports on abortion are provided to enable users to consider country policies in a broader health and human rights context.

To facilitate new insights on issues related to access and quality of care, all country laws and policies are juxtaposed to information from WHO guidance on safe abortion. The WHO safe abortion guidelines recommend that: “Laws and policies on abortion should protect women’s health and their human rights; Regulatory, policy and programmatic barriers that hinder access to and timely provision of safe services should be removed; An enabling regulatory and policy environment is needed to ensure that every woman who is legally eligible has ready access to safe abortion care; Policies should be geared to respecting, protecting, and fulfilling the human rights of women, to achieving positive health outcomes for women, to provide good-quality contraceptive information and services, and to meeting the particular needs of poor women, adolescents, rape survivors, and women living with HIV” [3].

In this paper we present the approach taken in the GAPD to coding and  classifying lawful abortion, the categories of abortion allowed or permitted by law or policy, and reflect on possible implications for women’s and girls’ health and rights and how increased transparency can facilitate state accountability for health and human rights in the context of abortion.

### Abortion laws and policies in a new light

The lawfulness of abortion - that is, the allowed or permitted categories of abortion as expressed through laws, policies, and guidelines - is a key component of the enabling environment for safe abortion. The continuum of lawful abortion ranges from abortion on a woman’s request with no requirement for justification, to specified grounds, to uncertain prohibition where laws prohibit unlawful abortion but do not specify any lawful grounds, to prohibition of all abortions. Where abortion is lawful, access can be restricted by gestational age, requirements for third-party authorisations, and an assortment of service-delivery requirements. The categories of lawful abortion, and how they are expressed in law and policy, have implications for who decides whether an individual is eligible and when, where, and by whom abortion services can be provided.

Efforts to categorize abortion laws and policies, and especially grounds for lawful abortion, began in the 1960s, resulting in the publication of several global databases in the mid-1970s and early 1980s [4–7]. The first UN abortion database was launched in 1987 with the sixth round of the *United Nations Inquiry among Governments on Population and Development* conducted by the UN Department of Economic and Social Affairs [8]. In 2001-2002 the UN published a three-volume global compendium of abortion policies*,* which included selected reproductive health indicators, accounts of abortion law reform, information on categories of lawful abortion and additional policies related to abortion access and service provision for 193 UN Member States [9]. A number of other organizations, including the Center for Reproductive Rights, the International Planned Parenthood Federation, the Sexual Rights Initiative, Women on Waves, and others also maintain databases on abortion laws and requirements for access to lawful abortion [10–13].

## Construction and content

### Coding and classification of laws and policies

To populate the GAPD a data extraction questionnaire was developed; an extensive search for source documents was conducted; and data were extracted, cross-checked, sent to countries for review, cross-checked again and uploaded. Coding in the GAPD is based on the explicit text of the law, policy, or guideline. The written words of legal text, however, take their meaning from their purpose and context and often require interpretation. Words may be interpreted narrowly or broadly, and may have different meanings depending on the context in which they are used. Local contexts and their legal systems will therefore always matter to the interpretation and meaning of an abortion law or policy. Nonetheless, all interpretations must start or engage with the text. The GAPD aims to add transparency about the lawfulness of abortion globally by focusing on the explicit text of the law, acknowledging that words found in laws are critical to interpretations that ultimately allow or deny access to abortion in practice.

Extracted data in the GAPD include abortion on a woman’s request with no requirement for justification, legal grounds, associated gestational limits, and legal requirements for access such as third-party authorisations, mandatory waiting periods, counselling and medical screening tests, and rules of insurance coverage. The GAPD treats legal grounds as the circumstances under which abortion is lawful, that is, allowed or not contrary to law, or explicitly permitted or specified by law. Common legal grounds for abortion include: to save a woman’s life; to preserve her health (often delineating physical and mental health); for economic and social reasons; in cases of intellectual/cognitive disability of the woman; and in cases of rape, incest and fetal impairment. When the law specifies a common legal ground, without qualification, the GAPD classifies it within that legal ground.

When the law specifies a legal ground differently from one of these common grounds, it is labelled as *other*. Examples of other grounds include the woman being below or above a specified age, contraceptive failure, unlawful sexual intercourse, and itemized lists of conditions and circumstances that may reflect more restrictive versions of legal grounds for saving life, preserving health, or economic and social reasons. 

Furthermore, some jurisdictions’ criminal code may have a medical or surgical treatment clause. These clauses exempt from criminal liability those who perform ‘in good faith and with reasonable care and skill a surgical operation upon an unborn child for the preservation of the mother’s life, if the performance of the operation is reasonable, having regard to the patient's state at the time, and to all the circumstances of the case’^3^ or deem abortion justifiable ‘for the purposes of medical or surgical treatment of a pregnant woman’^4^. Where such clauses refer to abortion in all but name and clearly state preservation of a woman’s life as the purpose, the GAPD classifies them as a legal ground to save the woman’s life.

To better understand the coding for abortion on a woman’s request and individual legal grounds in the GAPD, laws can be grouped into one of the following five categories:

1. Jurisdictions where the law prohibits all abortion;
2. Jurisdictions where *unlawful* abortion is prohibited or where there are only penalties for *unlawful* abortion, with no additional information provided about lawful abortion;
3. Jurisdictions where the law allows or  permits abortion only on one or more legal grounds;
4. Jurisdictions where the law entitles a woman to abortion on request with no requirement for justification, and where legal grounds may apply if a gestational limit for abortion on request is present and has been exceeded; and
5. Jurisdictions where abortion is regulated only as a health intervention in the health system.

In jurisdictions where laws prohibit abortion *except* or *unless* (or allow abortion *only if*) a legal ground or defence is specified, an ‘X’ is marked to reflect that abortion is unlawful on that particular ground and a check mark ‘✓’ indicates that abortion is lawful on that ground. In cases where only *unlawful* abortion is prohibited or penalised and it is unclear whether abortion is lawful on any particular ground, an ‘*i’* is marked and an accompanying note reflects that the legal classification in question is ‘not specified’.

### Regulations exist in different types of jurisdictions

A number of countries regulate abortion at the subnational and dependency levels. Traditional classifications of abortion laws typically only reflect national legislation and, in the case of countries where abortion is regulated at the subnational level, can appear to imply that laws in the most permissive jurisdictions apply nationwide. For example Mexico *Distrito Federal* allows abortion on a woman’s request, which is not permitted in the other 31 Mexican states. Thus, checking abortion on request for Mexico would inaccurately reflect abortion access for the majority of women and girls in the country. Reflecting differences in legal access at subnational and dependency levels highlights how women living in different geographic or administrative areas can have different degrees of access to lawful abortion, potentially leading to costly travel and associated opportunity costs as well as a higher likelihood of recourse to unsafe abortion where abortion is not allowed or permitted.

Globally, the total number of jurisdictions that regulate abortion is unclear. The GAPD currently includes all national jurisdictions but only selected subnational and dependency jurisdictions^5^ due to challenges associated with identifying, retrieving, and monitoring changes in laws and policies from provincial-, state-, and dependency-level authorities^6^. Although presently many subnational and dependency jurisdictions are not included in the database^7^, additions will be made as new information becomes available. All information for national, subnational, and dependency jurisdictions is current as of the date noted in the individual country profiles.

### Legal categories are treated as independent entities

By reflecting laws as written in the source documents, the GAPD treats each classification of lawful abortion independently – not as a subset of another – unless specified otherwise in the law. Thus, for example, it identifies jurisdictions that allow or permit abortion for health but not for life, rather than assume that abortion is also lawful to save the woman’s life; and the same criterion is applied to jurisdictions that allow abortion on a woman’s request but not for legal grounds of life, health, or fetal impairment.

For the purposes of classification, therefore, it is not assumed in the GAPD that a potentially broader ground such as health implies the existence of a narrower ground such as preservation of life. For example, in a country where lawful abortion is available only for a limited number of specified health conditions and there is no life ground, it is not assumed that a woman with a non-specified health condition leading to a fatal complication can lawfully access abortion services.

### Untested common law principles and other legal precedents

The common law doctrine of necessity stipulates that where abortion is illegal and an abortion is performed to prevent a greater harm such as death or severe harm to a woman’s health, the common law doctrine of necessity provides a legal justification or exculpation for the crime. However, in the GAPD, it is not assumed that abortion is permissible to save a woman’s life in jurisdictions where the common law doctrine of necessity *may* apply unless authoritative text – such as a Ministry of Health guideline or a court decision – confirms that the necessity defence is applicable to abortion.

In jurisdictions with a colonial past, data coding in the GAPD does not presume that pre-independence laws, regulations or legal precedents continue to apply unless there is evidence of their official adoption or application following independence. For example, in jurisdictions that are former colonies of the United Kingdom of Great Britain and Northern Ireland, it is not assumed that women and providers can rely on the 1938 judgment *Rex v Bourne*^8^ [14], which expanded access to abortion, in the absence of evidence of its official application.

## Utility and discussion

The GAPD shows that abortion regulation varies widely among countries – and within countries where abortion is regulated at subnational level – raising questions about the evidentiary and human rights basis for law and policy-making. For example, why set a gestational limit for abortion in cases of fetal impairment that is before a time when most fetal impairments are identifiable? Why allow abortion to save a woman’s life up to eight weeks gestation in one jurisdiction while there is no limit in others? Why set a limit for rape that is lower than for health when the psychosocial impacts of rape and a resulting pregnancy have clear health implications?

The GAPD highlights that in many jurisdictions legal and policy guidance is either non-existent, not clear, or contradictory. In these situations, when a woman seeks care and the medical professionals supporting her must determine whether she is lawfully entitled to have an abortion, they are compelled to rely on their own subjective, contextual interpretations of the relevant laws and policies. In the absence of legal clarity, women and providers may be confronted with the risk of potential investigation, prosecution and punishment, thus creating a chilling effect for women seeking, as well as health-care professionals providing, lawful services.

By juxtaposing a jurisdiction’s abortion laws and policies to relevant information and guidance from WHO and by facilitating comparisons of countries’ sexual and reproductive health indicators, the GAPD can enable deep policy analysis of states’ obligations to meet the health needs of individuals in the context of abortion. Furthermore, the United Nations Treaty Monitoring Bodies and Special Procedures now frequently publish their concerns and recommendations on abortion in concluding observations following periodic country reviews and country visits. The GAPD includes all concluding observations and Special Procedures reports that have addressed abortion since the year 2000. Policy analysis in the context of this authoritative human rights guidance can enable health and rights advocates to hold governments accountable for respecting, protecting, and fulfilling individuals’ human rights in the context of abortion.

## Conclusion

Abortion laws and policies can be punitive or protective, specific or non-specific, and limiting or facilitating for access and service provision. They are often found in a wide range of source documents, many of which are not readily accessible to the health-care professionals who must apply them, much less to the individuals who want to access services. Abortion laws and policies can be arbitrary, vague, confusing, and even contradictory, potentially exacerbating a chilling effect on those who seek, provide or advocate for access to services. Furthermore, laws can vary widely by jurisdiction, creating conditions for dramatic inequalities in access to safe, legal care. The Global Abortion Policies Database provides all of these details and more. It is a comprehensive tool that can generate legal knowledge, be used to inform law and policy research to examine the impacts of laws and policies in practice, and facilitate greater awareness of the many challenges to creating enabling policy environments for safe abortion.

## Abbreviations

EML: Essential medicines list
GAPD: Global Abortion Policies Database
HRP: UNDP-UNFPA-UNICEF-WHO-World Bank Special Programme of Research, Development and Research Training in Human Reproduction
UN: United Nations
WHO: World Health Organization

## Footnotes

1 Currently the GAPD contains no data for Equatorial Guinea, Democratic Peoples’ Republic of Korea, Federated States of Micronesia, Maldives, Marshall Islands, Niue, and St Vincent and the Grenadines.

2 Source documents in the GAPD are categorised under: reproductive health acts; general medical acts; constitutions; criminal/penal codes; ministerial orders/decrees; case law; health regulations/clinical guidelines; essential medicine lists (EML)/registered lists; medical ethics codes; documents relating to funding; abortion specific laws; laws on medical practitioners; laws on health care services; and other.

3 Surgical operations clauses exempting a woman from criminal liability are found in a number of countries in Africa and Oceania, including: Kenya, Malawi, Uganda, Kiribati, Nauru, Papua New Guinea, Solomon Islands, and Tuvalu.

4 Bahamas and Grenada are examples of countries that refer to ‘justification’ of abortion rather than exoneration from ‘criminal liability’ and do not refer to the aim of ‘preserving the mother's life’.

5 Subnational jurisdictions included in the GAPD: Australia – Australia Capitol Territory, New South Wales, Northern Territory, Queensland, South Australia, Tasmania, Victoria, and Western Australia; Bosnia and Herzegovina – Republika Srpska, Federation of Bosnia and Herzegovina; China – China (without Hong Kong), China-Hong Kong; Nigeria – Southern Nigeria, Northern Nigeria; Mexico – Mexico Distrito Federal; United Kingdom – (England, Scotland, Wales), Northern Ireland. Dependency jurisdictions include: United Kingdom – Guernsey, Isle of Man, Jersey.

6 In addition to the challenges of identifying, retrieving, and monitoring changes in subnational and dependency laws, collecting subnational (i.e., state-level) data from the United States of America presents a unique challenge associated with continuously changing state laws.

7 Currently, notable missing jurisdictions include 31 states in Mexico, 50 states in the United States of America, and a number of dependent territories, special administrative regions, and autonomous collectivities of Australia, China, Denmark, Finland, France, Netherlands, New Zealand, Norway, United Kingdom of Great Britain and Northern Ireland, United States of America, and perhaps other countries that have not yet been identified.

8 The 1938 ruling *R v Bourne* permitted abortion to prevent harm to a woman’s life and health.
